# Recent advances in unveiling active sites in molybdenum sulfide-based electrocatalysts for the hydrogen evolution reaction

**DOI:** 10.1186/s40580-017-0112-3

**Published:** 2017-07-25

**Authors:** Bora Seo, Sang Hoon Joo

**Affiliations:** 1 Department of Chemistry, Ulsan National Institute of Science and Technology (UNIST), 50 UNIST-gil, Ulsan, 44919 Republic of Korea; 2 School of Energy and Chemical Engineering, Ulsan National Institute of Science and Technology (UNIST), 50 UNIST-gil, Ulsan, 44919 Republic of Korea

**Keywords:** Hydrogen evolution reaction, Molybdenum sulfide, Electrocatalyst, Active site, Synthetic strategy, Structure engineering, Phase engineering

## Abstract

Hydrogen has received significant attention as a promising future energy carrier due to its high energy density and environmentally friendly nature. In particular, the electrocatalytic generation of hydrogen fuel is highly desirable to replace current fossil fuel-dependent hydrogen production methods. However, to achieve widespread implementation of electrocatalytic hydrogen production technology, the development of highly active and durable electrocatalysts based on Earth-abundant elements is of prime importance. In this context, nanostructured molybdenum sulfides (MoS_*x*_) have received a great deal of attention as promising alternatives to precious metal-based catalysts. In this focus review, we summarize recent efforts towards identification of the active sites in MoS_*x*_-based electrocatalysts for the hydrogen evolution reaction (HER). We also discuss recent synthetic strategies for the engineering of catalyst structures to achieve high active site densities. Finally, we suggest ongoing and future research challenges in the design of advanced MoS_*x*_-based HER electrocatalysts.

## Introduction

Hydrogen is a sustainable and renewable energy carrier that has demonstrated potential as an alternative to fossil fuel energy sources [1, 2]. Currently, hydrogen is produced mainly by steam methane reforming and coal gasification, leading to the ensuing problem of CO_2_ release [3, 4]. To provide a more environmentally friendly route to hydrogen power, the development of clean hydrogen production technology is required. Electrocatalytic water splitting represents the most promising solution for producing hydrogen using a carbon-free system [5, 6]. However, the dominant use of Pt-based catalysts for the hydrogen evolution reaction (HER) hinders the widespread implementation of electrocatalytic hydrogen production systems, due to their high costs and limited abundance. Hence, there have been significant efforts to replace Pt-based catalysts with highly active, durable, and non-precious electrocatalysts for the HER [7–19]. Notable examples of non-precious metal catalysts include metal sulfides, metal phosphides, metal carbides, and heteroatom-doped carbons. Among the various classes of non-precious metal-based electrocatalysts [7–82], nanostructured molybdenum sulfides (MoS_*x*_, *x* = 2–3) have been most widely studied, owing to their high activities, excellent stabilities, and precious metal-free compositions [7–68]. Although bulk MoS_2_ exhibits negligible catalytic activity for the HER [83], pioneering theoretical and experimental works by Nørskov and Chorkendorff have demonstrated that nanostructured MoS_*x*_ catalysts are able to catalyze the HER with high efficiency [20, 21]. During the last decade, significant progress has been made in designing MoS_*x*_ catalysts at the nanoscale, which has resulted in advanced MoS_*x*_-based catalysts with enhanced HER performances.

In this review, we highlight the key findings reported to date regarding identification of the active sites of MoS_*x*_ catalysts, and synthetic strategies for engineering their structures to yield high active site densities for the HER. Over the past decade, there have been a number of important developments relating to the active sites present in MoS_*x*_ catalysts. For example, the edge of MoS_2_ was first proposed as a catalytic active site by theoretical calculations in 2005 [20], which was later experimentally demonstrated with a model catalyst composed of MoS_2_ nanoparticles grown on a Au(111) surface [21]. Since then, various studies focused on maximizing active edge site densities via structural engineering approaches of MoS_*x*_ catalysts, including space-confined growth [22–25], vertical alignment [26–28], nano-assembly [29–31], and the design of biomimetic molecular catalysts [32–34]. The basal planes of MoS_2_, which were believed to be inert in the HER, have also been successfully activated to show meaningful activity by several strategies, including phase engineering from the 2H phase to the metallic 1T phase [26, 35–38], heteroatom doping [39–41], defect site generation [42–47], and strain engineering [40, 48, 49]. Despite significant investigations into the structural engineering of MoS_2_-based electrocatalysts to enhance the HER performance, a number of questions remain regarding the active sites and reaction mechanisms. For example, in the case of the amorphous MoS_*x*_, identification of its active sulfur sites for hydrogen adsorption has not yet been clarified due to its structural complexity [50–54].

In this review, we first discuss the basic concepts for the electrocatalytic production of hydrogen, in addition to the activity parameters commonly employed for evaluation of the HER activity. We highlight a number of important results regarding the active sites of MoS_*x*_-based HER catalysts, and summarize representative synthetic strategies for engineering their structures to enhance the number of active sites in different 2H-MoS_2_, 1T-MoS_2_, and amorphous MoS_*x*_ structures. We conclude the review by highlighting the current challenges and future research directions in relation to MoS_*x*_-based HER catalysts.

## Hydrogen evolution reaction

### Hydrogen evolution from water

The electrocatalytic production of hydrogen via water splitting is composed of two half reactions:

$$\begin{aligned} {\text{Anode}}{:} \, & 2 {\text{H}}_{ 2} {\text{O }} \leftrightarrow {\text{ O}}_{ 2} + {\text{ 4H}}^{ + } + {\text{ 4e}}^{ - } \left( {{\text{oxygen evolution reaction}},\,{\text{OER}}} \right) \\ & {\text{E}}_{\text{a}} = { 1}. 2 3 {\text{ V}} - 0.0 5 9\cdot{\text{pH }}\left( {{\text{V vs}}.{\text{ normal hydrogen electrode}},\,{\text{NHE}}} \right) \\ \end{aligned}$$

$$\begin{aligned} {\text{Cathode}}{:} \,& 4 {\text{H}}^{ + } + {\text{ 4e}}^{ - } \leftrightarrow {\text{ 2H}}_{ 2} \left( {{\text{hydrogen evolution reaction}},\,{\text{HER}}} \right) \\ &{\text{E}}_{\text{c}} = \, 0{\text{ V}} - 0.0 5 9\cdot{\text{pH }}\left( {{\text{V vs}}.\,{\text{NHE}}} \right) \\ \end{aligned}$$

**Fig. 1:**
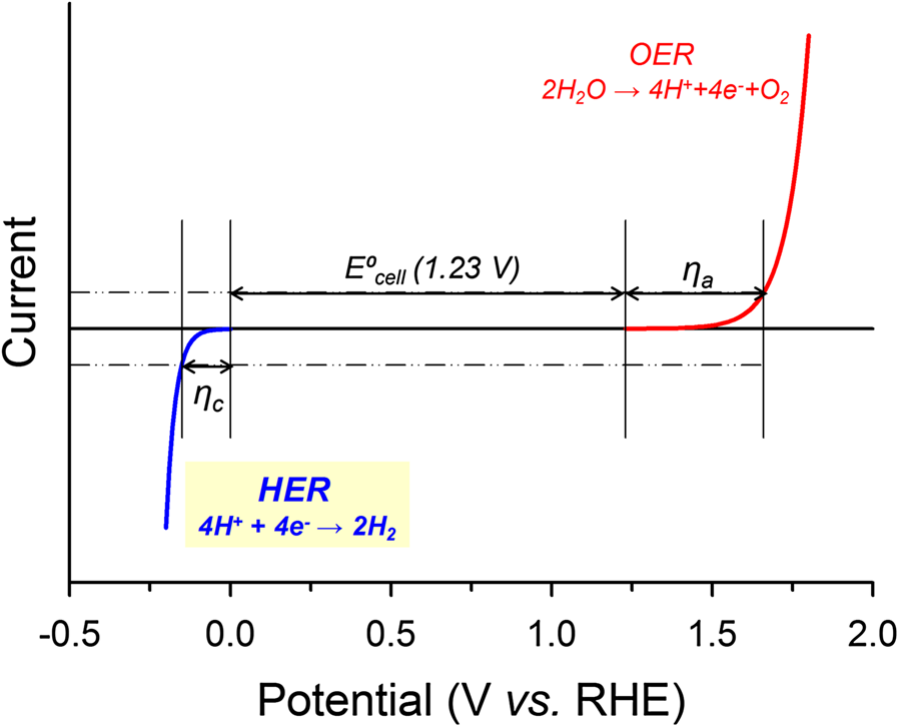
*I*-*V* curve for the full water splitting reaction

In this review, we will focus on the HER taking place at the cathode. Thermodynamically, the HER occurs with 0 V (vs. reversible hydrogen electrode, RHE) of applied potential, as shown in Fig. 1. However, for practical operations, a large excess potential, i.e., the overpotential (*η*_*c*_), is required, and the development of highly efficient HER electrocatalysts is directly linked to the reduction of the overpotential.

### HER activity parameters

#### Overpotential to drive a current density of −10 mA cm^−2^

Generally, the comparison of HER activities has been made in terms of the overpotential at a current density of −10 mA cm^−2^. This current density corresponds to an efficiency of approximately 10% in solar-to-fuel devices [84], and the derivation is as follows:

1. Integration of the solar spectrum (AM1.5G) yields a value of 100 mW cm^−2^, referred to as “1 sun”.
2. As the redox potential for water oxidation is ~1.2 V, an 100% efficient solar-to-fuel device would give 100 (mA V cm^−2^)/(1.2 V) = 83 mA cm^−2^ under AM1.5G.
3. Thus, a 10% efficient solar-to-fuel device would give 8.3 mA cm^−2^.

Therefore, the ranking of HER catalysts by comparison of the overpotentials required to drive a current density of −10 mA cm^−2^ is reasonable in a practical context.

#### Tafel slope

The Tafel slope is an important kinetic parameter, and is derived from the equation:

$$\left| \eta \right| = \frac{2.3RT}{\alpha nF}log\frac{J}{{J_{0} }}$$

where *η* is the overpotential, *R* is the ideal gas constant, *T* is the absolute temperature, *α* is the electrochemical transfer coefficient, *n* is the number of electrons involved in the electrode reaction, *F* is the Faraday constant, *J* is the measured current density, and *J*_0_ is the exchange current density. In addition, *J*_0_ is related to the electron-transfer rate of the reaction, reflecting the intrinsic catalytic activity of the catalyst. From the above equation, the Tafel slope is defined as $\frac{2.3RT}{\alpha nF}$ and bears the units mV dec^−1^. The Tafel slope is therefore determined from the linear regression line of the Tafel plots (*η* vs. *logJ*), which can be derived from the *I*-*V* polarization curve.

The Tafel slope has been used to access the HER mechanism taking place. More specifically, in an acid, the HER proceeds by the initial adsorption of a hydrogen atom on the catalyst surface $({\text{H}}_{{({\text{aq}})}}^{ + } + {\text{ e}}^{ - } \leftrightarrow {\text{H}}_{\text{ad}} )$, which is referred to as the Volmer step. Subsequently, molecular hydrogen is produced via the chemical recombination of two H_ad_ atoms (2H_ad_ ↔ H_2 (g)_; the Tafel step), or through a second electron transfer ($({\text{H}}_{{({\text{aq}})}}^{ + } + {\text{ H}}_{\text{ad}} + {\text{ e}}^{ - } \leftrightarrow {\text{H}}_{{ 2 { }({\text{g}})}}$; the Heyrovsky step) [7].

The HER following the Volmer–Tafel mechanism gives rise to Tafel slope of 29 mV dec^−1^, whereas the HER via the Volmer–Heyrovsky mechanism yields 38 mV dec^−1^. In both cases, the combination of two hydrogen atoms is the rate-determining step with lower value indicating faster reaction rate. When the Volmer step is the rate-determining step or the catalyst surface coverage is close to 1, the Tafel slope increases to 116 mV dec^−1^. The most of MoS_*x*_ catalysts have shown Tafel slopes in the range of 60–100 mV dec^−1^, following the Volmer–Heyrovsky mechanism.

#### Turnover frequency

The precise evaluation of each surface site’s activity is important to obtain a fundamental understanding of the origin of catalytic activity. The intrinsic activity can be assessed by calculating the turnover frequency (TOF), which is defined as the turnover rate per surface active site. While the comparison of TOFs is meaningful, a fair comparison of TOFs has not yet been carried out due to variations in methods for measuring the active sites in addition to the issues associated with different catalyst structures.

Depending on which sites are assigned as active centers, the TOF can be varied by many orders of magnitude. Theoretically, it is widely accepted that hydrogen atoms bind to surface S sites. However, the majority of studies have calculated TOFs by assuming that surface Mo atoms are the active sites, as the multiple chemical states for S render the calculation difficult. An alternative method is measuring an electrochemically active surface area (ECSA), which can probe all catalytically active surface sites, except the inert areas. Indeed, in some cases, ECSA-derived TOFs have afforded a fairer comparison between different electrocatalysts [80, 82].

It should be noted that the main active sites can differ according to the catalyst structure. For example, only edge sites are active in 2H-MoS_2_, while both edge and basal sites are active in 1T-MoS_2_. For amorphous MoS_*x*_ catalysts, the identification of active sites have been difficult due to complexity of their structures. Therefore, a standard method for TOF calculations should be established for fair comparison of MoS_*x*_ catalysts with different catalyst structures.

#### Gibbs free energy

The Gibbs free energy (∆*G*_H_) for atomic hydrogen adsorption has been widely used as an activity descriptor for HER catalysts. According to the Sabatier principle, particularly strong or weak interactions between reaction intermediates and catalysts can lower the overall catalyst efficiency. The HER activity therefore exhibits a volcano-shaped relationship as a function of ∆*G*_H_ [21]. Among the various catalysts examined, Pt catalyst exhibits the highest HER performance, with a ∆*G*_H_ value close to zero. It has been employed as a figure-of-merit for examining the HER performances of newly developed electrocatalysts. Generally, ∆*G*_H_ values are deduced by theoretical calculations using a simplified model to reflect the experimental conditions.

## Active sites in molybdenum sulfides

### Active edge sites

**Fig. 2:**
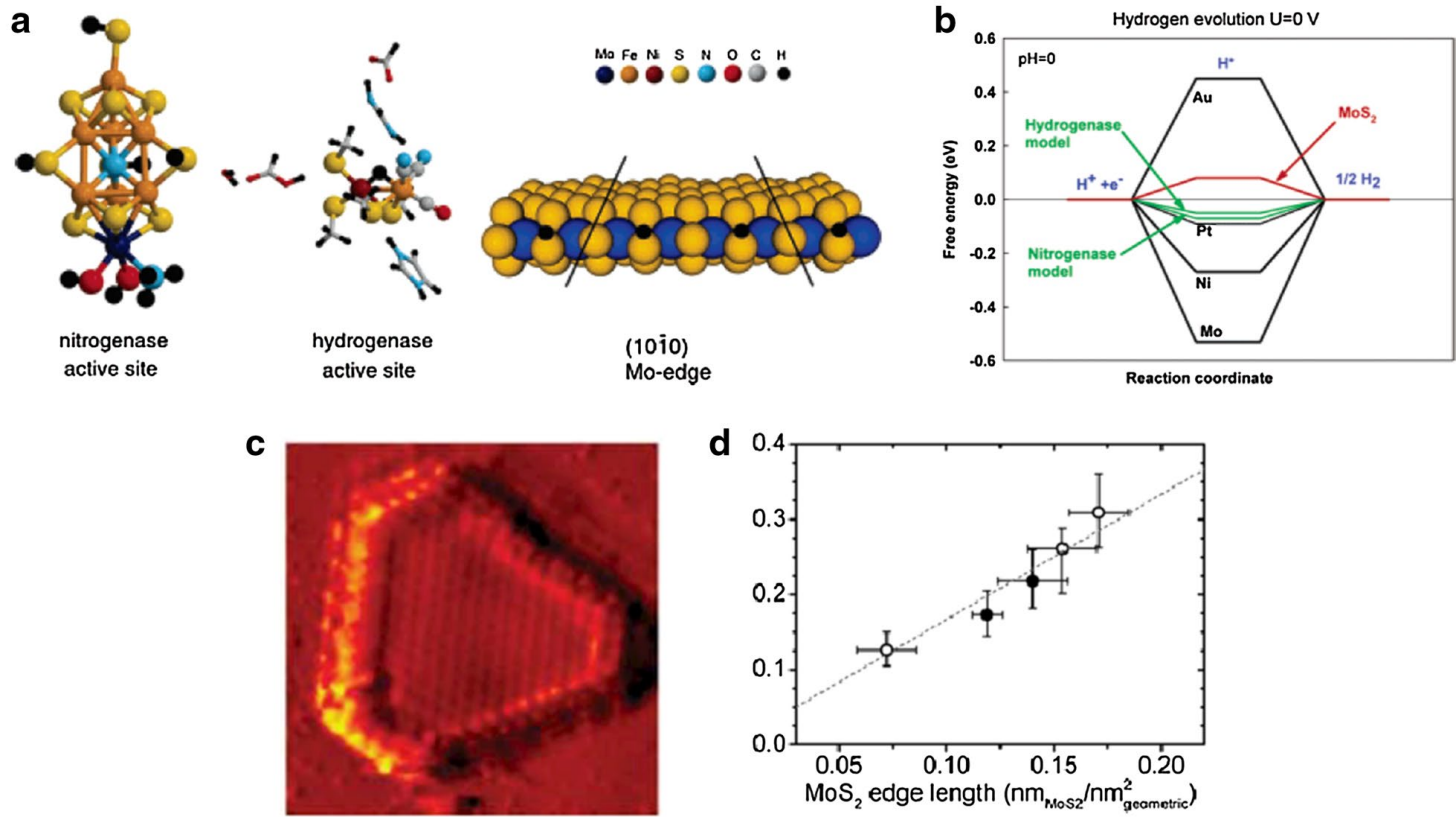
**a** Active sites of the nitrogenase and hydrogenase, and depiction of the Mo-edge on MoS_2_ slab. **b** Free energy diagram for hydrogen adsorption. **c** STM image of MoS_2_ nanoparticles on a Au(111) surface. **d** The exchange current density versus the MoS_2_ edge length (Figures reprinted with permission from Refs. [20, 21])

The active edge sites of MoS_*x*_-based HER electrocatalysts bear a remarkable resemblance to those of the nitrogenase and hydrogenase enzymes, which exhibit excellent activities and selectivities in their natural systems (Fig. 2a) [20]. To date, considerable progress has been made to synthesize highly active and stable heterogeneous electrocatalysts whose structures mimic the active sites of such natural catalysts [32–34]. In 2005, theoretical calculations by Nørskov and coworkers revealed that the ∆*G*_H_ value of the MoS_2_ edge is almost thermo-neutral, suggesting that the MoS_2_ edge is a highly plausible active site for the HER (Fig. 2b) [20]. Indeed, this was demonstrated experimentally using a model catalyst comprising MoS_2_ nanoparticles grown on a Au(111) surface (Fig. 2c) [21]. This work revealed that the electrochemical HER activity exhibits a linear correlation with edge length of MoS_2_ (Fig. 2d). These pioneering works have provided an important evidence that the active sites of MoS_2_ are located at the edge planes. Since these findings were reported, significant advances have been achieved in increasing the density of active edge sites [22–34], as will be discussed in Sect. 4.1.

Depending on the size of the MoS_2_ nanosheets employed, the edges of MoS_2_ can be covered with 0, 50, 75, or 100% sulfur atoms [55, 85, 86], where the sulfur coverage on the edges can significantly affect the adsorption of H atoms, which is directly correlated to HER kinetics. DFT calculations revealed that the most favorable edge configurations correspond to Mo edges covered by 50% S, where ∆G_H_ = 0.06 eV [87]. In addition to the sulfur coverage, the hydrogen coverage on the edges is also an important factor to determine the hydrogen binding energy [88].

### Active sites in 2H- and 1T-MoS_2_

**Fig. 3:**
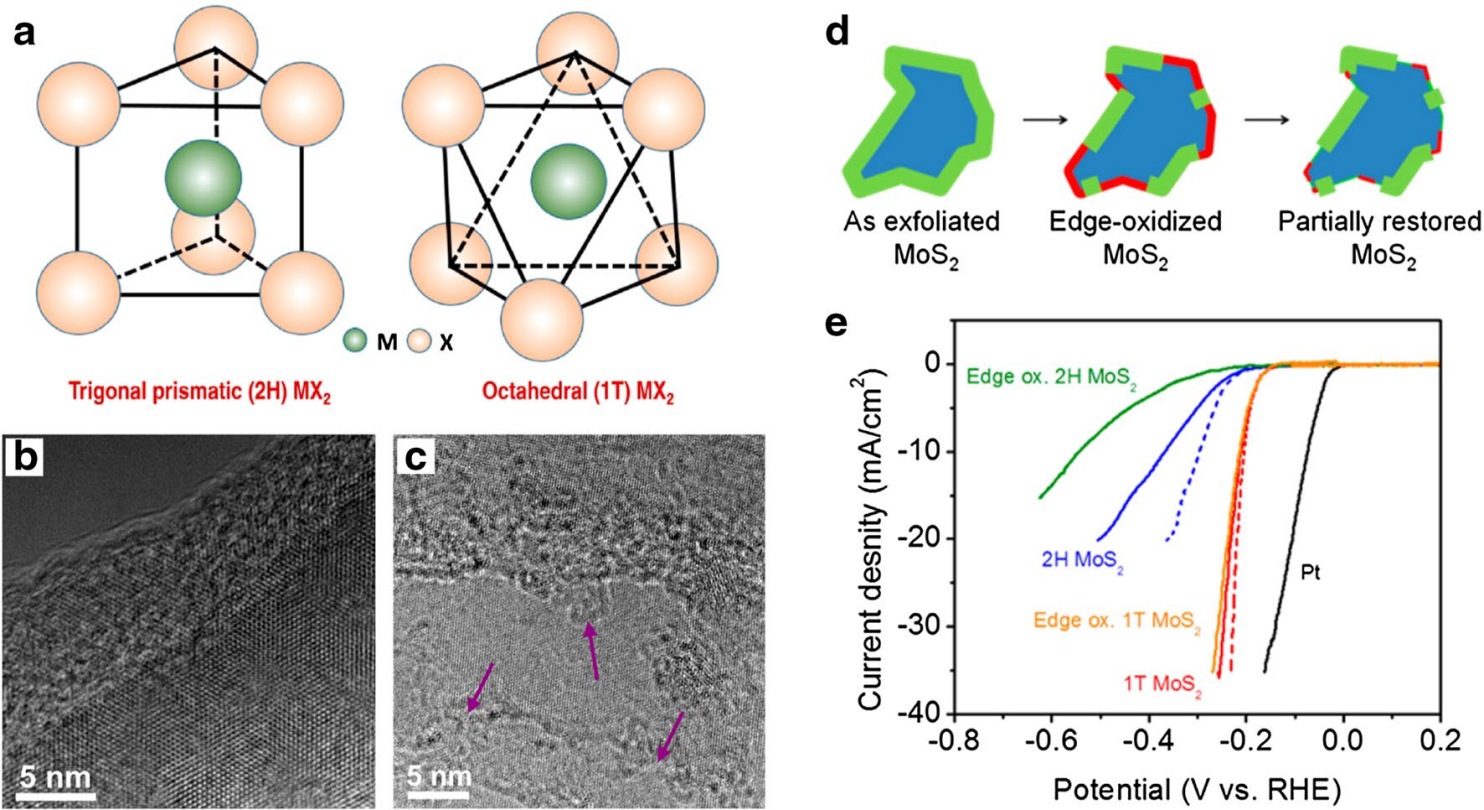
**a** Unit cell structures of 2H-MoS_2_ and 1T-MoS_2_. HRTEM images for **b** 2H-MoS_2_ and **c** 1T-MoS_2_. **d** Schematic representation of the oxidation process and partial restoration of the MoS_2_ edges after several voltammetric cycles. **e** HER polarization curves of 1T and 2H MoS_2_ before and after edge oxidation. *Dashed lines* indicate the iR-corrected polarization curves (Figures reprinted with permission from Refs. [17, 35, 36])

MoS_2_ has several polymorphs with distinct atomic configurations and electronic structures. Among these polymorphs, 2H-MoS_2_ and 1T-MoS_2_ structures are the most widely investigated for use as electrocatalysts in the HER. These polymorphs exhibit trigonal prismatic and octahedral unit cell structures, respectively (Fig. 3a–c) [17]. In addition, 1T-MoS_2_ has a dense atomic configuration in the basal surfaces and a high electronic conductivity, which is six orders of magnitude greater than that of 2H-MoS_2_, thereby resulting in an enhanced HER performance of 1T-MoS_2_ compared to 2H-MoS_2_. However, the preparation of 1T-MoS_2_ is more complicated than that of 2H-MoS_2_, due to its metastable nature. In this regard, significant efforts have been directed to prepare the stable and highly pure 1T-MoS_2_ polymorph using chemical Li-intercalation [35, 36], electrochemical Li-intercalation [26, 37], and a pressurized hydrothermal process [38]. For example, The Chhowalla group prepared the 1T phase MoS_2_ through an exfoliation reaction using lithium borohydride, where a significantly higher yield was obtained than that using n-butyllithium (i.e., 80% vs. ~50%) [36]. Using the exfoliated 1T-MoS_2_ sample, the main active sites of the two polymorphs, i.e., 2H-MoS_2_ and 1T-MoS_2_, were compared. Upon partial oxidation of the edges, the HER activity of 2H-MoS_2_ was significantly reduced, while that of 1T-MoS_2_ remained unaffected, suggesting that the main active site of the 2H-MoS_2_ polymorph is located at the edge sites, while that of 1T MoS_2_ may be located at basal sites. (Fig. 3d, e).

### Activating inert basal sites

**Fig. 4:**
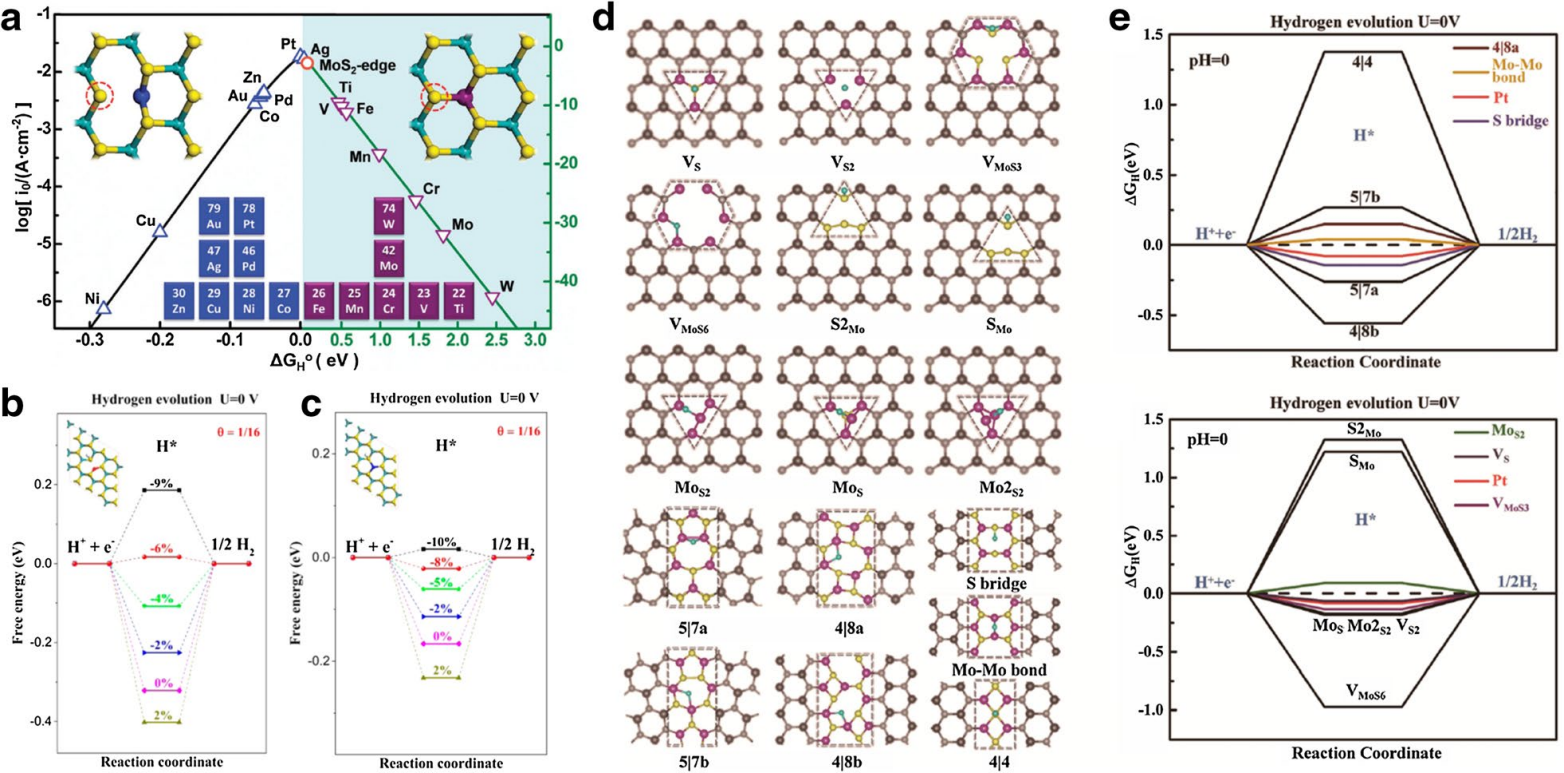
**a** The volcano-shaped relationship between the (log(i_0_)) and ∆*G*_H_. Free energy diagram for the HER on **b** Rh-doped MoS_2_, and **c** Ag-doped MoS_2_, at different strains. **d** 16 Examples of different structural defects. **e** ∆*G*_H_ of the HER process on each defect region (Figures reprinted with permission from Refs. [39, 40, 43])

The basal plane of the 2H-MoS_2_ polymorph has long been considered inert towards the HER, essentially rendering the large basal surface useless. However, several theoretical calculations have suggested that the inert basal planes of MoS_2_ can be exploited as potential active sites following activation by heteroatom doping, defect site generation, and strain engineering (Fig. 4) [39–49]. For example, heteroatom doping into MoS_2_ can produce high dopant concentrations on the surface, thereby modifying the hydrogen absorption strength of nearby surface atoms (Fig. 4a). More specifically, Du and co-workers suggested that doping MoS_2_ with a heteroatom in combination with a small compressive strain can yield an ideal ∆*G*_H_ for hydrogen binding in the HER (Fig. 4b, c) [40]. In addition, defects are known to perturb the local density of states, creating additional energy levels below the conduction bands [89]. In this context, Wang and co-workers evaluated the effect of sixteen different structural defects on activating the basal plane of MoS_2_ monolayers (Fig. 4d) [43]. The theoretical results suggested that six defects, including sulfur vacancies, greatly enhanced the HER performance of MoS_2_ (Fig. 4e). These theoretical findings were later verified experimentally, as discussed in Sects. 4.3 and 4.4.

### Active sites in amorphous MoS_*x*_

**Fig. 5:**
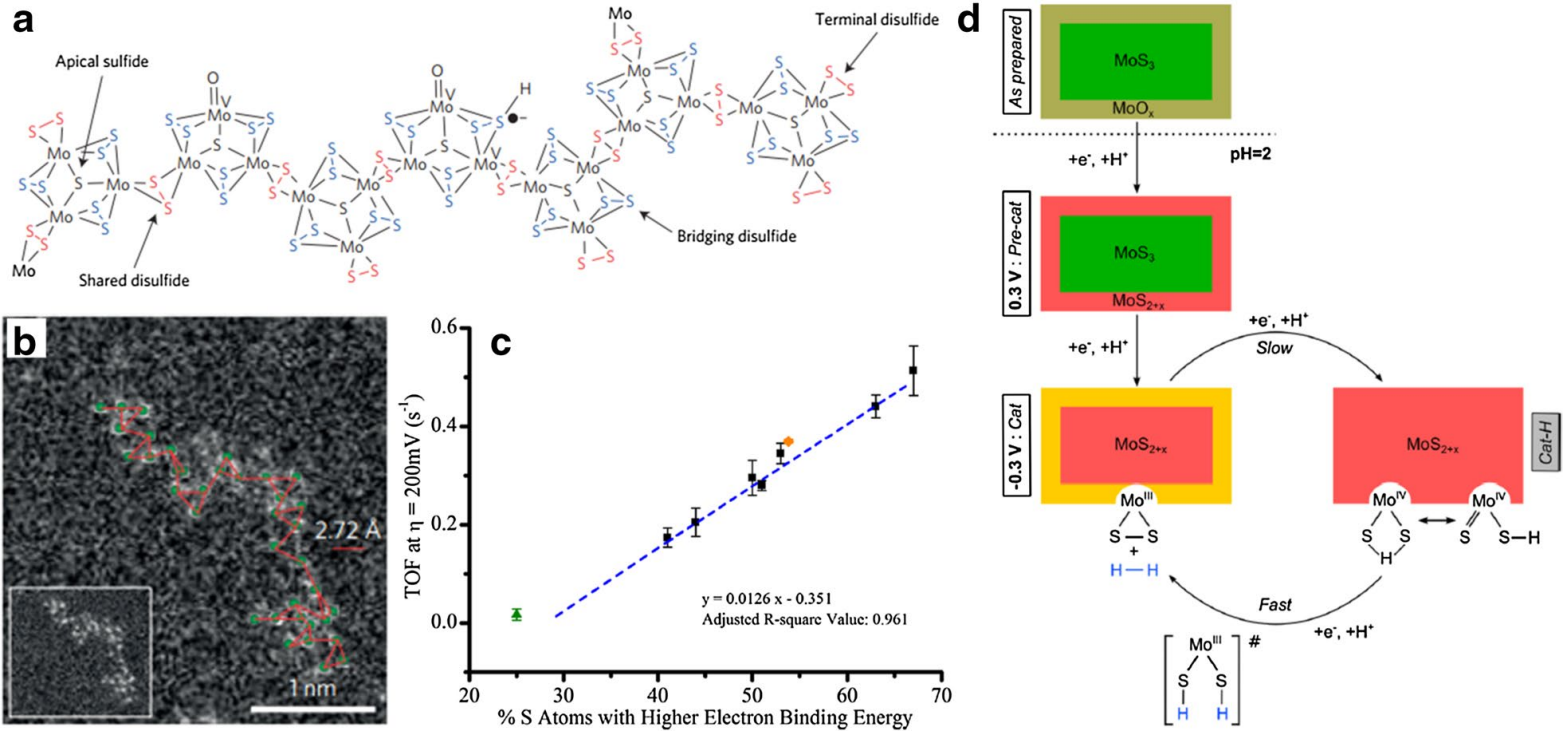
**a** Coordination polymeric structure of the amorphous MoS_*x*_ containing [Mo_3_S_13_]^2−^ building blocks. **b** Arrangement of the cluster units in a 1D unfolding chain. **c** TOFs versus the percentage of S atoms with high electron binding energy. **d** Proposed catalytic cycle for the HER over amorphous MoS_*x*_ (Figures reprinted with permission from Refs. [51–53])

In addition to the crystalline MoS_2_ structure, amorphous MoS_*x*_ has attracted significant attention due to its facile preparation under mild conditions, such as wet chemical synthesis [60] and electrodeposition [58, 59, 61]. Unlike MoS_2_, the active sites present in amorphous MoS_*x*_ have received little attention due to the complex polymeric structure of such compounds (Fig. 5a) [52]. It contains short-range atomic arrangements with [Mo_3_S_13_]^2−^ clusters as building units (Fig. 5b). Similar to MoS_2_, the question of active sites on amorphous MoS_*x*_ has been unavoidably and continuously raised. Recently, the sulfur atoms present in amorphous MoS_*x*_ have been directly confirmed as the catalytic active sites for the HER via operando Raman spectroscopic analysis [54]. However, the sulfur atoms exist in four key states, namely bridging S_2_^2−^, terminal S_2_^2−^, unsaturated S^2−^, and apical S^2−^. Due to the diverse sulfur chemical states present in the amorphous MoS_*x*_, identification of the catalytically active sulfur sites for proton reduction is challenging. Interestingly, Yeo and co-workers reported a linear correlation between TOFs for the HER and the percentage of S species with higher electron binding energies using X-ray photoelectron spectroscopy (XPS) (Fig. 5c) [53]. This work suggested bridging S_2_^2−^ species as the potential catalytic active sites. In addition, Yano and Hu and co-workers investigated the structural changes taking place in the amorphous MoS_*x*_ under HER conditions using in situ X-ray absorption spectroscopy (Fig. 5d) [51]. They proposed a reaction mechanism, where the catalytic species is similar to MoS_2_, which corroborates an earlier result by Nilsson and Jaramillo and co-workers [50]. Although significant efforts have been devoted to revealing the active sites for proton reduction, the identification and confirmation of genuine catalytically active sulfur sites remain elusive.

## Synthetic strategies for increasing active site densities

### Nanospace-confined growth

**Fig. 6:**
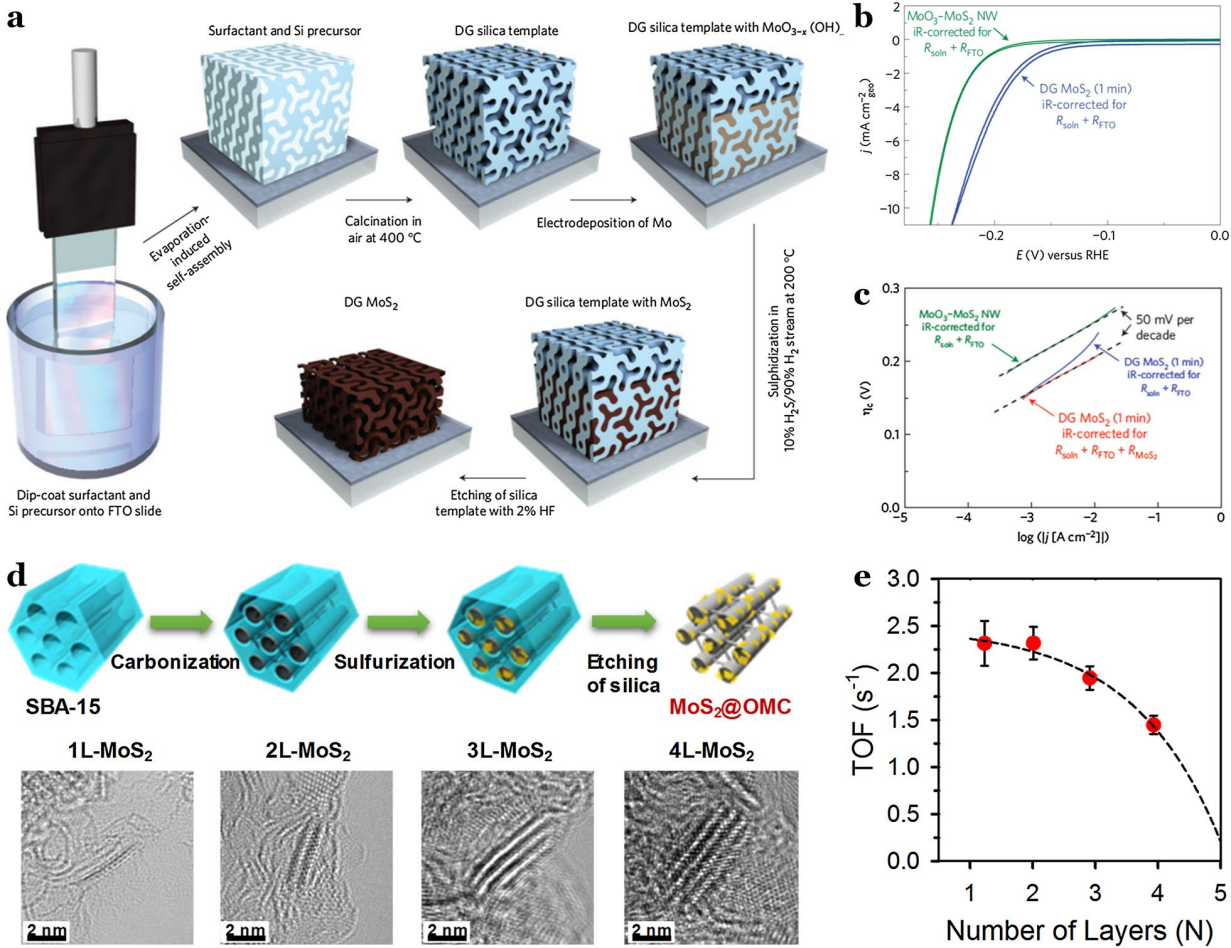
**a** Synthetic procedure and structural model for the mesoporous MoS_2_ with a double-gyroid (DG) morphology. **b** Cyclic voltammogram of DG MoS_2_ (1 min sample) versus core–shell MoO_3_-MoS_2_ nanowires (NW) at 5 mV s^−1^. **c** Tafel plot of DG MoS_2_ (1 min sample) versus core–shell MoO_3_-MoS_2_ NW. **d** Synthetic procedure for the MoS_2_ nanosheets embedded on ordered mesoporous carbon nanorods (MoS_2_@OMC), and TEM images for 1L-, 2L-, 3L-, and 4L-MoS_2_@OMC. **e** TOFs versus the number of layers in the MoS_2_ (Figures reprinted with permission from Refs. [22, 23])

Reducing the particle size of MoS_2_ is the most straightforward method that can increase the density of active edge sites. However, thermodynamics tends to favor growth through the basal plane because the formation of edge sites is highly energetic due to the under-coordinated atomic configuration. To overcome this challenge, confinement growth within a nanospace has been reported [22–25]. The Jaramillo group successfully synthesized a mesoporous MoS_2_ structure using a silica template with double-gyroid (DG) morphology (Fig. 6a) [22]. The resulting DG MoS_2_ structure exhibited a high surface curvature, thereby exposing a large fraction of active edge sites. The DG MoS_2_ exhibited a higher HER performance than high aspect-ratio core–shell MoO_3_–MoS_2_ nanowires (Fig. 6b). In addition, the DG MoS_2_ gives a Tafel slope of 50 mV dec^−1^, which is relatively low compared to previously reported MoS_2_-based HER catalysts (Fig. 6c). In this direction, our group prepared layer number-controlled MoS_2_ nanosheets using the nanospace confined-growth approach [23]. In this work, the pores of mesoporous silica templates were partially filled with carbon, and MoS_2_ structures were subsequently grown inside the residual nanospace of the silica-carbon composites. After the etching of silica template, MoS_2_ nanostructures embedded in the frameworks of ordered mesoporous carbons (MoS_2_@OMC) were generated. As shown in the TEM images (Fig. 6d), the formation of an extended basal plane was successfully hindered with a size <5 nm in the lateral direction. It was found that the TOF increased upon decreasing the layer number in MoS_2_, and this trend in activity could be correlated to the physical and chemical properties of MoS_2_ nanoplates (Fig. 6e). These results indicate that space confinement growth paves the way to controlling the surface structure and size of MoS_2_ at the nanoscale to ultimately develop effective catalysts with high densities of active edge sites at the surface.

### Phase engineering

**Fig. 7:**
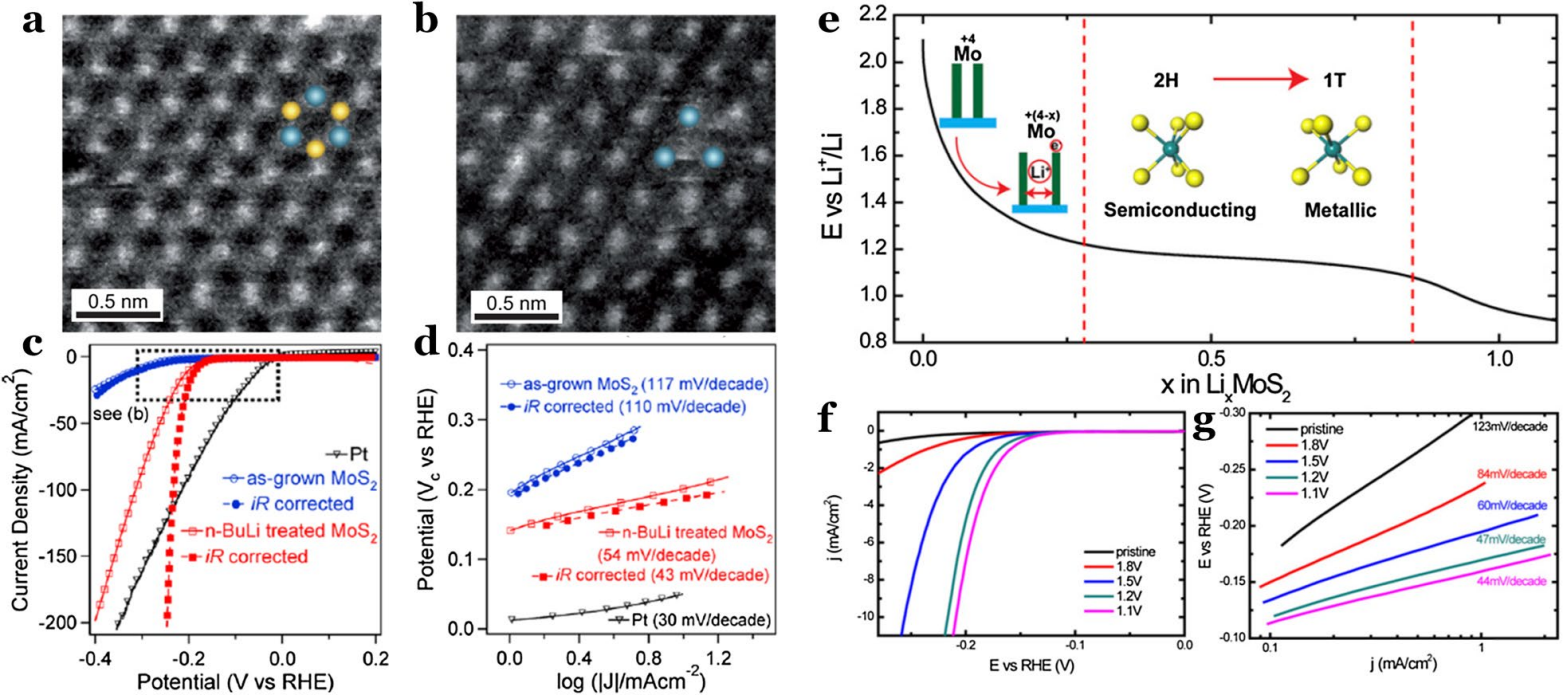
Dark-field scanning transmission electron microscopy images of **a** 2H-MoS_2_, and **b** 1T-MoS_2_. **c** Polarization curves of the 2H- and 1T-MoS_2_ for the HER, and **d** the corresponding Tafel plots. *Filled symbols* indicate the iR-corrected data. **e** Galvanostatic discharge curve representing the lithiation process. **f** Polarization curves of the pristine and lithiated MoS_2_ for the HER, and **g** the corresponding Tafel plots (Figures reprinted with permission from Refs. [9, 26, 35])

As mentioned in Sect. 3.2, the active sites, which were limited to the edges in 2H-MoS_2_, have been expanded to basal surfaces via phase engineering from trigonal prismatic (2H-MoS_2_) to metallic octahedral (1T-MoS_2_) structures (Fig. 7a, b). For example, the Jin group demonstrated that the chemical exfoliation of 2H-MoS_2_ using n-butyllithium significantly enhances the HER activity through the formation of metallic 1T-MoS_2_ [35]. This phenomenon was ascribed to the fast electron transport and increased number of active sites guaranteed by the metallic 1T-MoS_2_ nanosheets. These 1T-MoS_2_ nanosheets required a low overpotential of 187 mV to drive a current density of −10 mA cm^−2^, compared to ~313 mV for 2H-MoS_2_ (Fig. 7c). Furthermore, the Tafel slope of 43 mV dec^−1^ for 1T-MoS_2_ was significantly lower than that of 2H-MoS_2_ (i.e., 110 mV dec^−1^), indicating fast HER kinetics in 1T-MoS_2_ (Fig. 7d). As an alternative phase engineering technique, the Cui group reported the use of an electrochemical Li intercalation method to generate the 1T-MoS_2_ phase (Fig. 7e) [26]. This method allowed the vertically-aligned 2H-MoS_2_ to be converted into 1T-MoS_2_, which exhibited an enhanced HER performance (Fig. 7f, g). In addition to the intercalation of Li ions, mechanical strains also induced the partial formation of 1T-MoS_2_ structures, thereby activating the HER [67]. However, despite numerous reports focusing on the use of phase engineering to enhance HER performances, the origin of the HER activity has not yet been completely elucidated. Decoupling of the intrinsic activities of 1T phase from the overall HER activity is required to reach a fundamental understanding of the active sites present in MoS_2_ polymorphs.

### Heteroatom-doping

The incorporation of heteroatoms into the basal surface of MoS_2_ nanosheets can significantly modify the electronic structure of in-plane S atoms neighboring the heteroatom, thereby altering the adsorption strength of H atoms. In this context, the Bao group reported the doping of single Pt atoms into the in-plane domain of MoS_2_ nanosheets (Pt-MoS_2_) [39], where the resulting Pt-MoS_2_ exhibited an enhanced HER performance compared with the undoped MoS_2_. Furthermore, they also screened the HER activities of MoS_2_ doped with a number of transition metals, resulting in volcano-shaped relationships with the adsorption free energy of the H atoms (∆*G*_H_) (Fig. 4a). Their study suggests a novel method for activating the inert in-plane domain of MoS_2_ catalysts, which may also be extended to other 2D materials applicable in a variety of catalytic reactions.

### Defect and strain engineering

**Fig. 8:**
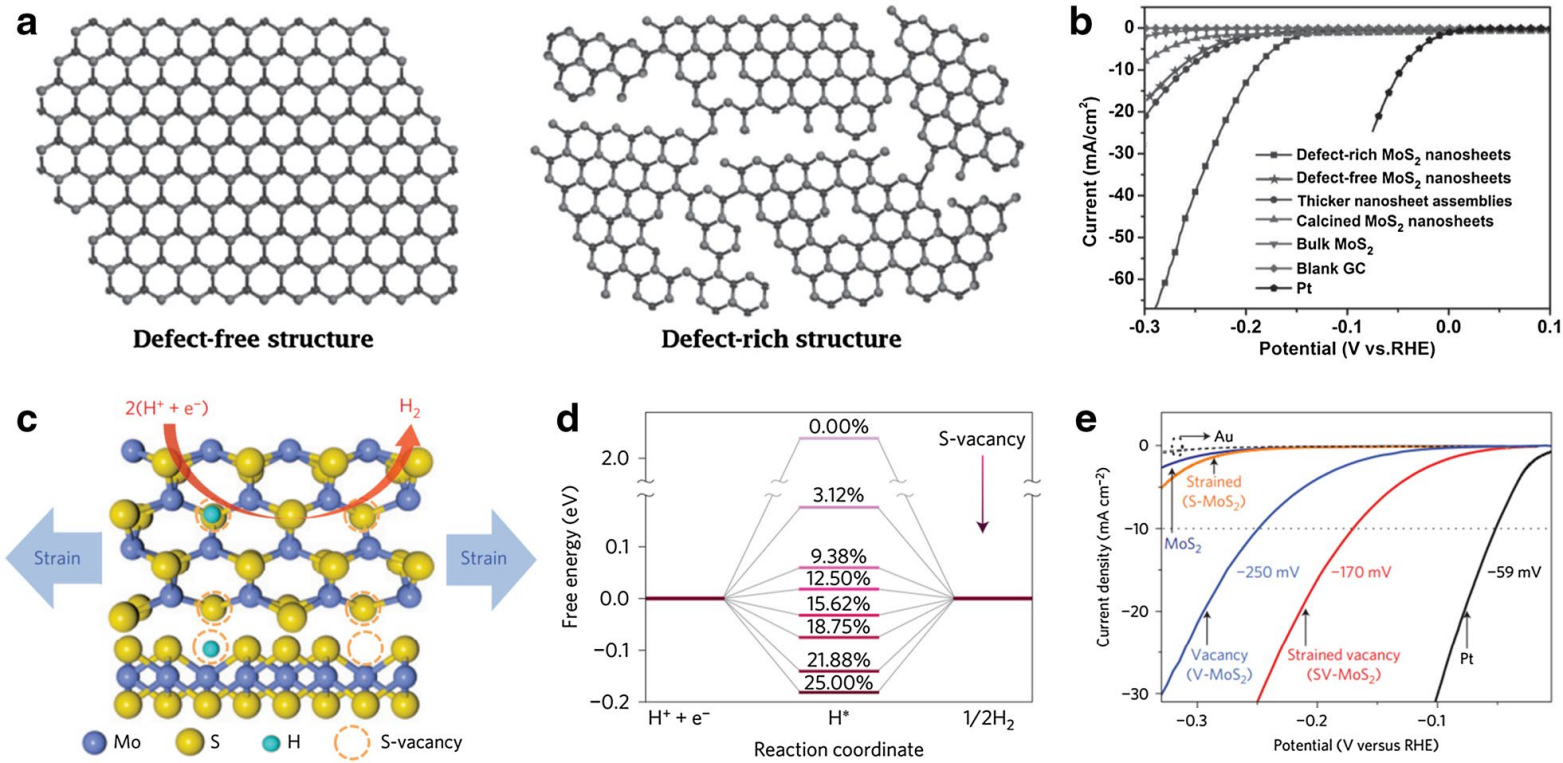
**a** Structural models of the defect-free and defect-rich MoS_2_ structures. **b** Polarization curves of the defect-free and defect-rich MoS_2_ structures for the HER. **c** Schematic representations of the *top* and *side* views of MoS_2_ containing strained S-vacancies on the basal planes. **d** Free energy versus the HER reaction coordinate for the S-vacancy range of 0–25%. **e** Polarization curves for the strained, vacancy, and strained vacancy MoS_2_ for the HER (Figures reprinted with permission from Refs. [42, 48])

The inert basal surfaces of 2H-MoS_2_ have also been successfully activated by creating defect sites and/or inducing strain [42–47]. The first example of defect engineering conducted by Xie and co-workers focused on the exposure of additional active edge planes by forming cracks on the surfaces of nanosheets (Fig. 8a) [42]. They reported that defect-rich MoS_2_ exhibited a significantly enhanced HER performance compared with defect-free MoS_2_ (Fig. 8b). In addition, the Ajayan group demonstrated that oxygen plasma treatment and H_2_ annealing introduced additional active sites within the MoS_2_ monolayer, significantly improving the HER activity [44]. Recently, more rational and controllable defect modulation has been reported through combined experimental and theoretical studies [45–47]. Allwood and co-workers prepared MoS_2_ nanocrystals and activated the Mo atoms in the basal surface of MoS_2_ nanocrystals by S depletion [45], with the resulting activated MoS_2_ exhibiting an very high HER performance (~150 mV at −10 mA cm^−2^ and a Tafel slope of ~29 mV dec^−1^). Cao and co-workers also verified the importance of S vacancies on the catalytic activity for the HER [46], estimating the intrinsic TOFs of the edge sites, S vacancies, and grain boundaries as approximately 7.5, 3.2, and 0.1 s^−1^, respectively. Finally, the Zheng and Nørskov groups reported that straining of the S-vacancies further enhances the HER activity (Fig. 8c–e) [48]. The experimental results was further verified with theoretical results that optimum level of strain and S-vacancy can tune the Δ*G*_H_ close to zero, guaranteeing the highest intrinsic HER activity.

## Summary and future challenges

In this review, we have highlighted recent major achievements regarding elucidation of the active sites in MoS*x*-based electrocatalysts for the HER, and summarized synthetic strategies for designing MoS_x_ electrocatalysts with enhanced HER activities. The edge site of MoS_2_ was initially identified as the active site for the HER, which then triggered the development of new HER catalysts that can maximize the density of active edge sites, thereby boosting HER activity. The basal surface of MoS_2_, which were previously believed to be inert towards the HER, can be converted into HER active species via appropriate structural engineering, which include phase transformation, heteroatom doping, defect site generation, and strain engineering. In addition to the crystalline MoS_2_, amorphous MoS*x* has also been extensively studied as an efficient electrocatalyst for the HER. Amorphous MoS*x* can possess abundant active edge structures originating from the building blocks of [Mo_3_S_13_]^2−^ clusters, however, the multiple chemical states of sulfur in such species hamper identification of the actual active sulfur states. A comprehensive and systematic study to reveal the key active sites in different MoS*x* structures still remains a challenging task. Understanding of active sites would enable high-performance MoS*x* elecrocatalysts for the HER.
